# Maskless, Reusable Visible-Light Direct-Write Stamp for Microscale Surface Patterning

**DOI:** 10.1021/acsami.2c20568

**Published:** 2023-02-17

**Authors:** Tomas Javorskis, Tomas Rakickas, Alberta Janku̅naitė, Šaru̅nas Vaitekonis, Artu̅ras Ulčinas, Edvinas Orentas

**Affiliations:** † Department of Nanoengineering, Center for Physical Sciences and Technology, Savanorių 231, LT-02300 Vilnius, Lithuania; ‡ Department of Organic Chemistry, Vilnius University, Naugarduko 24, LT-03225 Vilnius, Lithuania

**Keywords:** surface patterning, photoacids, photolithography, soft lithography, catalytic lithography, surface functionalization, HD-DVD laser

## Abstract

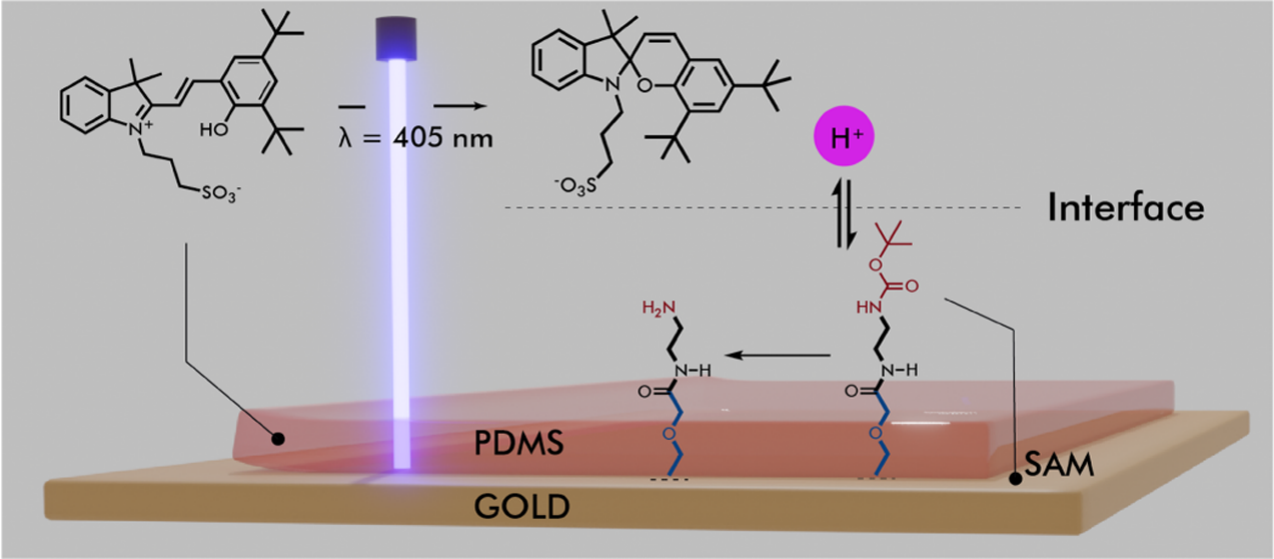

We report a straightforward method for creating large-area, microscale resolution patterns of functional amines on self-assembled monolayers by the photoinduced local acidification of a flat elastomeric stamp enriched with photoacid. The limited diffusivity of the photoactivated merocyanine acid in poly(dimethylsiloxane) (PDMS) enabled to confine efficient deprotection of *N*-*tert*-butyloxycarbonyl amino group (*N*-Boc) to line widths below 10 μm. The experimental setup is very simple and is built around the conventional HD-DVD optical pickup. The method allows cost-efficient, maskless, large-area chemical patterning while avoiding potentially cytotoxic photochemical reaction products. The activation of the embedded photoacid occurs within the stamp upon illumination with the laser beam and the process is fully reversible. Preliminary positive results highlight the possibility of repeatable use of the same stamp for the creation of different patterns.

## Introduction

Surfaces modified with physically or chemically addressable components rank among the best platforms to investigate the interfacial phenomenon and to create functionally relevant devices, such as sensors,^1^ (bio)chips for biomedical applications,^2–4^ information storage,^5^ drug discovery,^6^ or diagnostics.^7^ Most meaningful applications of functional surfaces, however, require the creation of patterns of various geometries, the dimensions and chemical nature of which are dictated by the intended function. Nanoscale patterning, for instance, is highly sought-after for future electronic,^8,9^ information storage,^5^ and nanoelectromechanical systems (NEMS)^10^ to maximize the density of working components, whereas in other fields, especially those focused on cell–interface interactions, micrometer patterning is sufficient or even desired.^2,3^ The choice of particular patterning method is dictated by the type of the surfaces; thin films of polymeric material are usually very robust and tolerate a wide range of chemical or physical treatments,^11−13^ while molecule-thick self-assembled monolayers (SAMs) are much more delicate.^10,14^ Despite their high sensitivity, SAMs, on the other hand, provide highly ordered surfaces of perfectly controlled thickness, surface functionality, and density and are prepared easily from molecularly well-defined and synthetically modifiable components, such as thiols.^14,15^ The development of robust, easy-to-implement, and scalable surface patterning techniques applicable to SAMs is challenging but central to the advancement of material science.

Among numerous methodologies developed for large-area, (sub-)micrometer feature size chemical patterning, probably the most common are photolithography^16^ and microcontact printing.^17,18^ Both of these approaches in their traditional implementations require predefined physical information carriers, such as photomasks or stamp masters, with the shape of the desired features encoded into them. While perfectly suitable for many recurring tasks, they can become cumbersome when arbitrary features on the surface are required. Therefore, direct-write or maskless approaches continue to draw considerable research effort.

Approaches most commonly used for direct-write chemical patterning of SAMs can be assigned to two broad categories: use of the energetic beam(s) to achieve a local SAM decomposition or desorption (e.g., direct laser writing^19^ and electron beam lithographies^20^) and scanning probe-based methods, where a small probe is used to deliver material or energy, which leads to the chemical changes in SAM.^13,21−24^ While features down to the range of several nanometers can be readily fabricated using these methods, the equipment cost for the former and the limited throughput for the latter remain a challenge for large-area patterning.

For chemical patterning of surfaces, the amino functionality is among the most versatile ones due to its easy modifications with a variety of protecting groups or well-established amide bond formation. The chemistry of the amine group on the surface is very robust and to date has been extensively used.^25−31^

The most general and straightforward method to pattern free amino groups on the surface would require a suitable protecting group (PG) that could be removed by a localized reagent. Photocleavable PG would be ideal as it requires no chemicals for the deprotection and utilizes easily controllable light stimulus.^32,33^ However, in this case, photosensitive PG has to be installed on SAM and upon cleavage would generate various byproducts. Residues of such chemical waste might be detrimental to biological applications. Indeed, most photocages for amino group protection contain nitroaromatic^34−36^ or coumarin derivatives,^37^ both having high intrinsic toxicity. In addition, photocaged SAM would be difficult to handle and would have a limited shelf-life because of its light sensitivity.

Among chemically addressable PG, the acid-sensitive *N*-*tert*-butoxycarbonyl (*N*-Boc) group is widely used in chemistry, including surface modification.^18,26,38^ The Boc-cleavage reaction produces traceless gaseous CO_2_ and isobutylene and can be performed catalytically. Nevertheless, for efficient deprotection, very corrosive acids such as trifluoroacetic or hydrochloric acids are used. When applied to SAM, chemical damage and desorption of thiols are often observed. Although surface patterning using these reagents is hardly possible, acidic polyurethane stamps with surface-confined sulfonic acid have been successfully applied for microcontact printing on SAMs.^39^

Recently, we have reported on-demand patterning of Boc-terminated SAMs using heat and local delivery of water to achieve localized cleavage of the protective Boc group.^40^ However, the achieved resolution was in the order of hundreds of micrometers, which is too low for many potential applications. Here, we describe a new method of direct-write, photocatalytic patterning for the creation of amine-terminated patterns on Boc-protected aminothiol SAMs. The technique utilizes flat and nonpatterned elastomeric stamps enriched with photoacid, which upon illumination with focused visible (λ = 405 nm) light lead to a local cleavage of the Boc group at room temperature. The elastomeric nature of the stamp ensures reliable conformal contact with the SAM-decorated surface, permitting uniform large-area patterning. By virtue of employing the focused light for acid photoactivation and limited diffusivity of activated acid, spatial confinement of deprotected areas with a resolution better than 10 μm is achieved. This approach permits the creation of arbitrary patterns of active amines on the surface suitable for further direct (bio)chemical functionalization. Importantly, the chosen reaction route avoids the formation of potentially toxic reaction products and is therefore particularly attractive for biological applications.

## Experimental Section

### Materials

Ethanol (99.8%, Honeywell) was distilled and stored in a glass bottle prior to use. Ultrapure water (resistivity 18.2 MΩ·cm at 25 °C) was directly used from a Synergy 185 UV water purification system (Merck KGaA, Germany). Nitrogen gas, purity of 99.999% (N_2_, ElmeMesser Lit, Lithuania), was used for sample drying. Qdot545 ITK Streptavidin Conjugate Kit (Qdot545) and Qdot655 Biotin Conjugate Kit (Qdot655) were obtained from ThermoFisher Scientific. Substrates for experiments were cut from gold-coated silicon wafers (with a 20 nm thick Au film and a 2 nm Ti adhesion layer, Ssens BV, The Netherlands). PDMS stamps were prepared using prepolymer and curing agent (10:1 ratio w/w, Sylgard 184 kit, Dow). A self-assembled monolayer was formed using thiols prepared by previously reported synthesis.^40^ Biotin-PEG_4_-NHS was purchased from Merck, Germany, and used without additional purification. The chemical structure of the derivatization reagent (Biotin-PEG_4_-NHS) is given in Figure S1. Photoacid was synthesized according to the procedure reported by Zayas et al.^41^

### SAM Formation

Before modification, gold-coated substrates were sonicated (in an ultrasonic bath, RK100H, Sonorex, Bandelin, Germany) in distilled ethanol twice for 1 min and washed in the SC-1 solution (a mixture of ultrapure water, 30% hydrogen peroxide (Carl Roth GmbH, Germany), and 25% ammonia solution (Carl Roth GmbH, Germany) in 5:1:1 v/v/v, respectively) at ∼75 °C for 5 min. Then, they were thoroughly rinsed in ultrapure water and dried under an N_2_ gas stream. Immediately after cleaning, the substrates were immersed in a thiol solution, containing 5% mol of C_16_EG_4_NHBoc (T_1_) and 95% mol C_16_EG_1_OH (T_2_) with a total concentration of 100 μM in distilled ethanol and incubated for at least 14 h to form SAM. After incubation, the substrates were rinsed and sonicated twice in distilled ethanol for 1 min and dried with the N_2_ gas.

### Photoacid-Enriched Stamp Preparation

Sylgard 184 kit was used for standard stamp preparation. Prepolymer and curing agent (10:1 ratio w/w) were mixed and the solution of photoacid in dichloromethane (2.5 mM) was added in portions while mixing (3.0 mL of solution for 2 g of PDMS), stirred until dichloromethane evaporated, and degassed in vacuum for 20 min. Next, this mixture was spin coated on fluoro-silane-coated Si substrate forming a ∼35 μm layer that was cured for 1 h at 65 °C. Afterward, clean PDMS was poured over the photoacid-enriched layer to mechanically reinforce it. Final curing for 13 h at 65 °C provided a ∼0.5 mm thick PDMS stamp. Before use, this PDMS stamp was sonicated in ethanol for 5 min and dried in a desiccator under reduced pressure for 0.5 h.

### Patterning with HD-DVD Pickup

The experimental setup was built around the HD-DVD optical pickup (OPU; PHR-803T, Toshiba, Japan) controlled by an in-house-developed and built electronic controller (see the Supporting Information for more details). As the absorbance of the photoacid used in this work is located primarily in the blue region, only the blue (λ = 405 nm) laser beam was employed. For patterning, PA-enriched stamps were placed on SAM-containing substrates, the beam was focused through the photoacid-enriched stamp on the gold surface on which the SAM was formed, and the sample was illuminated with controlled power and duration. Sample positioning and translation in XY were implemented by two motorized positioning stages (8MT167-25, Standa, Lithuania) and computer-controlled using software developed in-house.

### SAM Derivatization with Biotin-PEG_4_-NHS

The deprotected substrate after sonication in ethanol (3×) and H_2_O (1×) was immersed in 1.0 mL of PB buffer (pH 8.0) and the solution of Biotin-PEG_4_-NHS (1.5 mg/200 μL H_2_O) was added in one portion. After 15 min, the substrate was washed with H_2_O and additionally sonicated in H_2_O and chloroform.

### Visualization of Deprotected Areas

Two systems were used for visualization, Qdot545 (streptavidin-coated Qdots) and Qdot655 (biotin-coated Qdots). The protein and quantum dot solutions were dissolved in the HEPES buffer (20 mM HEPES, 150 mM NaCl, 0.001% Tween 20, pH 7.4). Fluorescence images were recorded with an Olympus BX51 upright microscope, equipped with 10× NA 0.3 and 100× NA 1.0 water immersion objectives, an epi-fluorescence unit, and a mercury lamp (Olympus, Tokyo, Japan). Images were acquired with a Peltier-cooled F-View II CCD camera (Olympus Soft Imaging Solutions GmbH, Münster, Germany) and analyzed using the StreamMotion software (Olympus Soft Imaging Solutions GmbH, Münster, Germany). This microscope was also equipped with a motorized sample stage (Prior Scientific Instruments Ltd., United Kingdom), which was used to construct the composite images of areas larger than the field of view of the respective objectives. The lines appearing in the large-area composite images at the borders of the stitched images result from the uneven illumination in the field of view. When using Qdot545, the sample was immersed in 400 μL of HEPES buffer and the Qdot545 solution (2 μM, 0.5 μL) in the HEPES buffer (100 μL) was added. After incubating for 15 min, the sample was washed with HEPES. When using Qdot655, the sample was immersed in 400 μL of the HEPES buffer and the avidin solution (45 μM, 1 μL) in the HEPES buffer (100 μL) was added. After 15 min, the incubation sample was washed with HEPES buffer and the Qdot655 solution (2 μM, 0.5 μL) in the HEPES buffer (100 μL) was added. After incubating for 15 min, the sample was again washed with HEPES buffer.

## Results and Discussion

To combine the advantages of the *N*-Boc group availability and the operational convenience of light stimulus, we devised a method to reversibly perform the localized activation of acid triggered by blue light. For this purpose, metastable photoacid (PA) based on the merocyanine/spiropyran photoswitch was selected.^42^ These PAs are widely used in various applications and can achieve large differences in effective p*K*_a_ between the spiropyran (SP) and merocyanine (MC) forms.^43−45^ Physical embedment of PA in the PDMS matrix would thus allow localized proton generation in bulk and on the surface. On contact with *N*-Boc-protected SAM, the photogenerated pattern would be directly transferred to a surface as deprotected areas. In contrast to chemically amplified resists, where a photoacid generator is mixed within the active layer, the contact mode deprotection eliminates the chemical contamination of the SAM. Moreover, the activation of merocyanine photoacid operates in the visible region as opposed to the UV light necessary for chemically amplified resists and, most importantly, requires no mask. Finally, the use of reversible acid photoactivation would in principle allow the recycling of the stamp for multiple uses.

**Figure 1:**
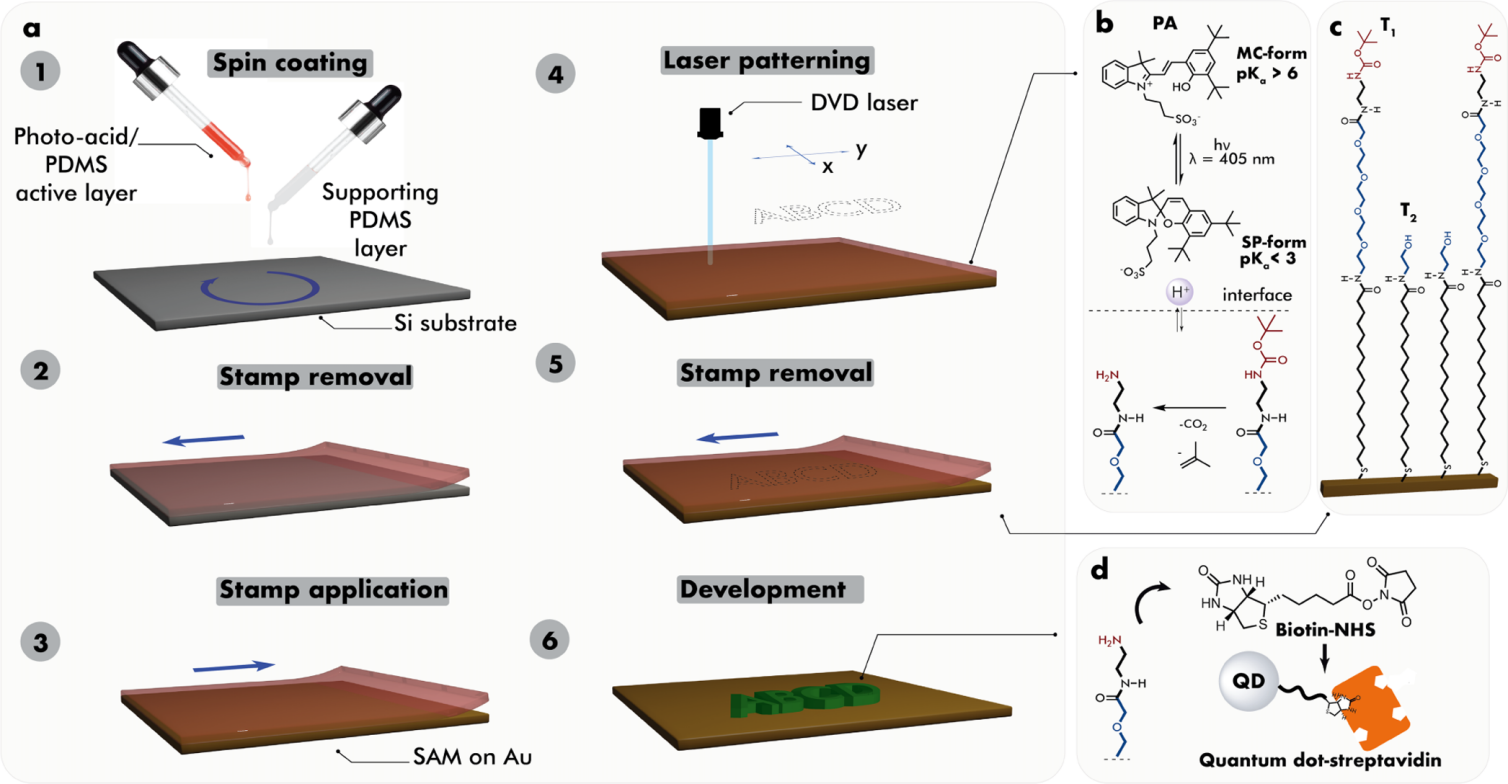
(a) Representative scheme for surface patterning. The working PDMS layer (∼35 μm) containing photoacid is spin-coated on the Si substrate followed by the supporting layer of pure PDMS (step 1). The stamp is removed from the substrate (step 2) and applied onto the surface of the SAM (step 3). Laser patterning is accomplished by irradiation with a blue laser of the HD-DVD optical pickup using a computer-generated image (step 4). The visualization of the pattern was performed by removing the photoactive stamp (step 5) and developing first with biotin-NHS and then with quantum dot-streptavidin reagents (step 6). (b) Chemical structures of merocyanine (MC) and spiropyran (SP) forms of the photoacid (PA) and the corresponding deprotection reaction at the interface. (c) Chemical structures of working T_1_ and supporting T_2_ thiols within the SAM. (d) Two-step functionalization of deprotected amines.

The full patterning procedure is schematically depicted in Figure 1a. Photoacid PA appended with bulky *tert*-butyl groups was chosen because of its low dark acidity, fast reversion kinetics to the nonacidic state after the end of light exposure, and excellent solubility in nonpolar solvents (Figure 1b).^41^ The PDMS prepolymer and the curing agent were mixed with the dichloromethane solution of PA and spin coated on a flat silicon substrate (Figure 1a, step 1) and cured. The concentration of PA in the PDMS stamp was optimized to be the highest possible, near the saturation level where the crystallization of PA was observed. The obtained photoactive layer was approximately 35 μm thick. It was additionally strengthened by casting a thicker transparent layer of a clean PDMS layer (approximately 0.5 mm) over the photoactive part. After the second curing, the obtained stamps were peeled off the silicon substrate and washed by ultrasonication in EtOH before further use (Figure 1a, steps 1 and 2). The stamp was next brought into conformal contact with the SAM surface (Figure 1a, step 3). The SAM is composed of two thiols T_1_ (5%) and T_2_ (95%) of varying lengths. The longer-working thiol T_1_ features the hydrophobic mercaptohexadecanoic acid connected to the hydrophilic tetraethylene glycol unit via an amide bond (Figure 1c). The thiol is terminated with the *N*-Boc-protected ethylene diamine segment. The amide bond connecting the hydrophilic and hydrophobic parts ensures lateral aggregation through hydrogen bonds resulting in highly ordered SAM.^46,47^*N*-Boc-terminated thiols without reinforcing amide groups are known to form loosely ordered low-density SAMs.^23^ The shorter thiol T_2_ is complementary to T_1_ at the amide connector but contains only a single ethylene glycol unit at its terminus. Mixing of working thiol with a shorter thiol was necessary to dilute the bulky *N*-Boc groups to reduce surface hydrophobicity and minimize the nonspecific adsorption of protein reagents used in a subsequent derivatization step.

For patterning, a blue laser diode (BLD) beam with controlled power of the HD-DVD optical pickup^48−50^ was used to illuminate the PA-enriched stamps placed on SAM-containing substrates to initiate the generation of the photoacid. To transfer the designed digital pattern, the sample is translated by XY-motorized stages in a computer-controlled fashion. The *N*-Boc deprotection reaction takes place at the illuminated regions after photoinduced cyclization of PA and concomitant local acidification at the surface of the stamp (Figure 1a, step 4). Following the illumination and resulting deprotection of SAM, chemical derivatization was used to visualize areas of free amine groups (Figure 1a, steps 5 and 6). For this purpose, we used an amide bond formation reaction with an *N*-hydroxysuccinimide-activated biotin linker following the treatment of derivatized areas with streptavidin-coated quantum dots for fluorescence imaging (Figure 1d).

**Figure 2:**
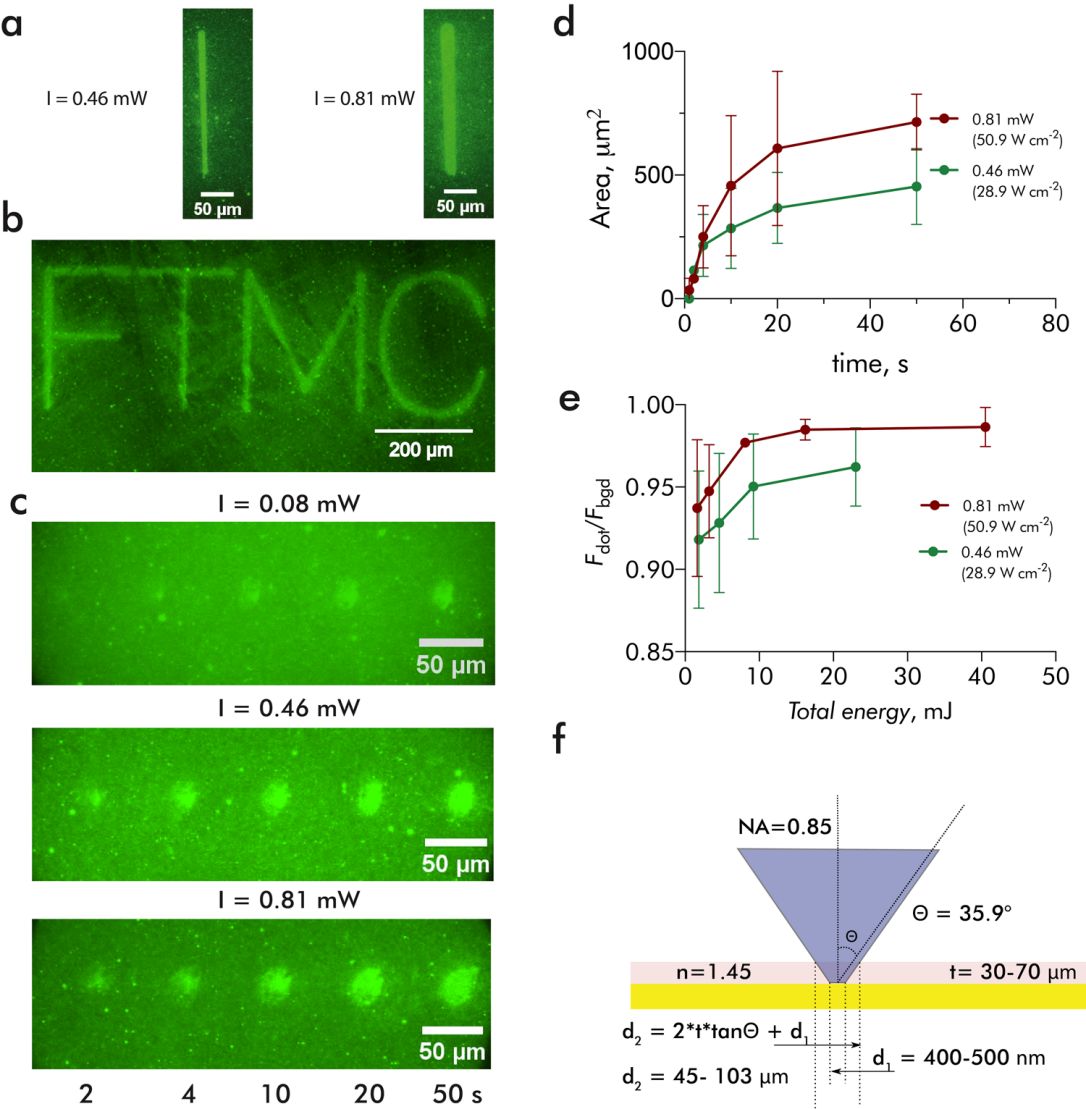
(a) Rectangular patterns produced at different illumination powers. The scanning rate is 25 μm/s; (b) pattern of letters; (c) area of dot patterns as a function of illumination time at various illumination powers; (d) area of dots as a function of the illumination time; (e) fluorescence intensity as a function of the total energy; and (f) scheme of the focused beam with dimensions at the top and the bottom of the active PDMS layer.

Figure 2a shows the fluorescence patterns of deprotected SAM produced by rectangular scans of focused BLD at two different illumination powers and a constant writing rate. The shape of the scan was well reproduced in the deprotected areas, indicating the ability to attain spatial control of the deprotection process by the focused beam. A more complicated letter pattern was also cleanly produced (Figure 2b). To ensure that the areas containing deprotected and reactive primary amines are visualized by the biotin-streptavidin-QD derivatization, the following control experiment was carried out. The step involving biotinylation of the deprotected sample was omitted, proceeding instead directly to treatment with streptavidin-conjugated QDs. In this case, no biotin binding to the primary amines via amide bond takes place, and streptavidin-conjugated QDs would be attached to the surface only due to the nonspecific adhesion. However, no fluorescent areas were observed, confirming that no significant nonspecific attachment of QDs due to laser-induced SAM degradation or other factors took place. We further demonstrated the ability to controllably functionalize deprotected areas of SAM with protein by derivatizing the biotinylated areas with avidin, followed by visualization by biotin-conjugated QDs (see the Supporting Information). Furthermore, simple irradiation of SAM through a PA-free PDMS stamp results in no fluorescent image corroborating the importance of acid for the deprotection step.

We found that the best resolution affording adequate patterning speed and pattern clarity was 8.0 μm for the translation velocity of 25 μm s^–1^ at 0.46 mW (28.9 W cm^–2^). This is comparable to the other studies that reported resolutions from tens^19^ to several^1,51^ to 1 μm^52^ for photolithography-based approaches to pattern nonspecific protein adsorption-resistant films. SNOM-based methods permit another order-of-magnitude improvement in resolution,^24^ albeit at the cost of the increased complexity of the equipment and patterning procedure.

The dimensions of the deprotected areas are somewhat larger than the requested scan dimensions even when taking into account the broadening due to the finite size of the beam. The line broadening increases with illumination power (Figure 2a). We hypothesized that this discrepancy is caused by the diffusion of the photoactivated acid in the PDMS stamp and perhaps even at the stamp–SAM interface. To check this assumption, we investigated the deprotection process using stationary illumination with the focused beam with varying power and duration. Figure 2c shows dot patterns obtained after the visualization of the deprotected areas. Dimensions of the dots as well as fluorescence intensity exhibit dependence on the power and duration of illumination. We calculated the areas of the deprotected dots using the thresholding filter and particle analysis; the corresponding graph is shown in Figure 2d. Figure 2e shows the relative fluorescence intensity of the deprotected dots (which is proportional to the deprotection efficiency) scaled by the average background intensity as a function of total illumination energy. Both dependencies reach saturation at longer illumination durations and reveal no straightforward dependence on illumination energy.

We assume that the processes of acid photoactivation and diffusion of proton-containing species determine the final dimensions of the deprotected dots and the deprotection efficiency. Since the activation of the acid is proportional to the illumination energy, the diffusion of acid (protons) is also enhanced by the illumination power. The decrease of pattern resolution due to acid diffusion has also been reported for molecular glass resists and showed identical saturation behavior.^53,54^ Although the broadening effect might be a result of the diffusion of PA from the stamp onto the SAM surface, other factors related to the finite thickness of the active layer might also be at work. As shown in Figure 2f, the focused beam of BLD has dimensions in the 400–500 nm range at the focal point; however, the exposed volume within the PDMS layer is significantly higher. Simple derivation based on the numerical aperture of the lens and the typical thickness of the layer (30–70 μm) gives an estimate of the diameter of the illuminated region at the top of the PA-enriched layer in the range of 45–103 μm. Diffusion of the acid from the illuminated conical region toward the SAM surface is likely accountable for pattern broadening. Previously reported stamps with covalently attached sulfonic acids produced identical patterns regardless of the stamping time, confirming a critical role of such a diffusive spread.^39^ The saturation of the diffusive spread can be explained by the consumption of PA at the trapping sites of the liberated amino groups or ethylene glycol units of the supporting thiol or might simply reflect the depletion of the PA at the irradiation cone. The control experiments also revealed that a 0.46 mW illumination power is optimal to provide comparable deprotection efficiency as the highest energy beam, but potentially less harmful to photoacid and SAM.

**Figure 3:**
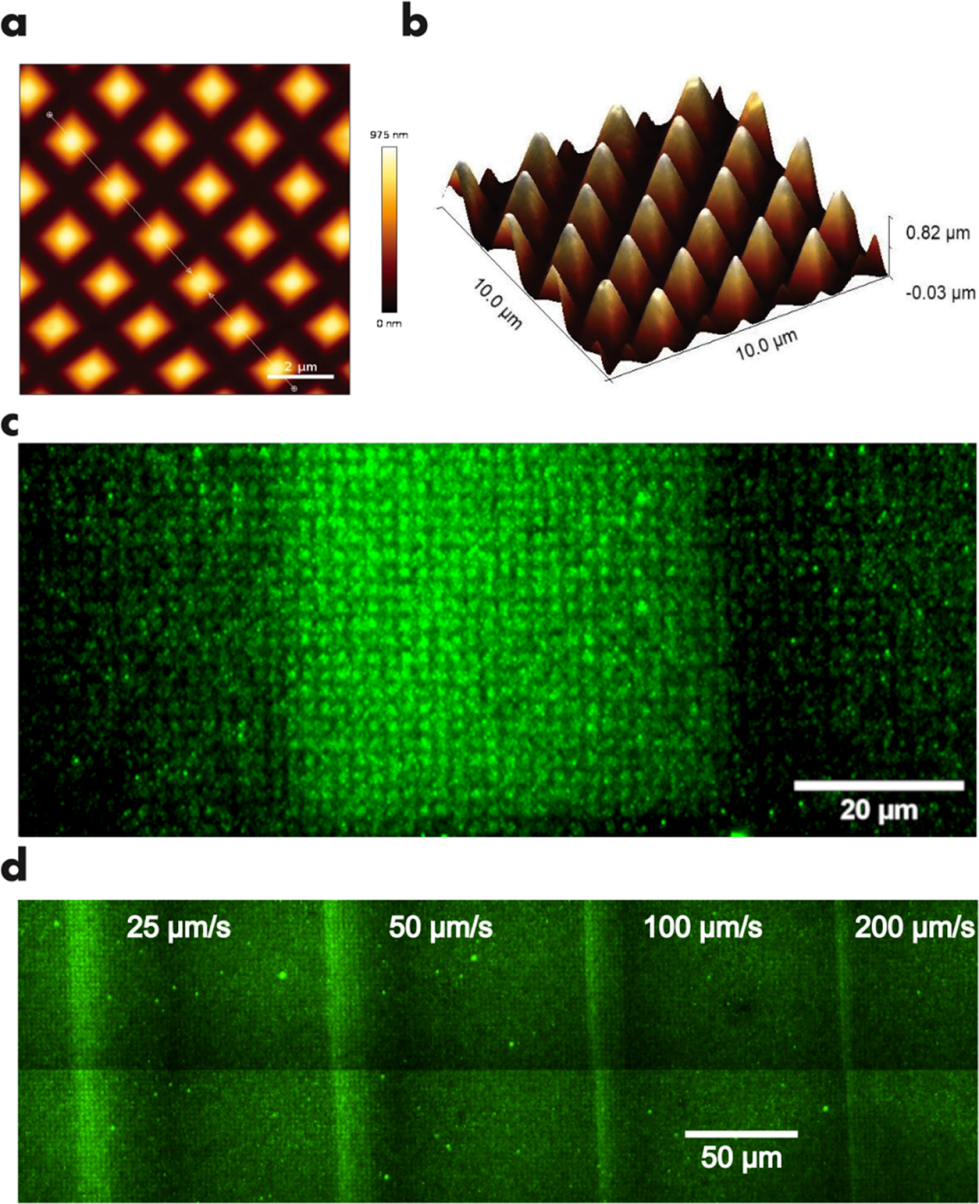
Microstructured PA-enriched PDMS stamp. (a) Atomic force microscopy height image of the stamp surface. (b) 3D representation of the height data in (a). (c) Fluorescence image of the area deprotected using a microstructured stamp and blue laser diode activation (0.46 mW). (d) Lines produced at various writing rates.

Having established that diffusion of photoacid in the PA-enriched PDMS stamp plays an essential role in determining the final dimensions of the deprotected areas, we investigated the possibility to limit this undesired process without resorting to a more complex process involving the covalent fixation of PA. For this, we have manufactured PA-enriched PDMS stamps having microstructured pyramidal protrusions, with the aim to reduce both volume available for PA diffusion and the stamp–SAM interfacial contact area. The approach is similar to the strategy used in beam pen lithography where an array of pyramidal structures fabricated in an elastomer is used as individually addressable tips for delivering molecular ink, or for focusing light to localize the photochemical reaction, except that the pyramids were smaller.^55−57^ The PA-enriched PDMS stamp was manufactured by casting the PDMS–PA mixture using Klarite (SERS substrate with inverted pyramids) as a replica template (Figure 3a,b). Figure 3c,d show the fluorescence image of the deprotected areas obtained by scanning the focused beam in a line pattern at constant power while varying the scanning rate (see the Supporting Information). It is evident that the pyramidal microstructure of the PA-enriched stamp was reproduced as an array of square-shaped dots, with dimensions corresponding to those of the base of the pyramidal protrusions. The sharp tips of the pyramids evidently have been compressed under the weight of the stamp. However, no broadening beyond the dimensions of the pyramids was observed, confirming that the deprotecting action of the PA was indeed limited to the areas of direct interfacial contact between the stamp and the SAM. The broadening of the lines beyond the dimensions of the focused beam, again, is likely a product of the diffusion of the photoacid in the PDMS volume and not on the SAM surface. Applying different scanning rates for line patterning, inverse dependence of line thickness vs scanning rate was observed (Figure 3d). Shorter exposure times of PA at a higher scanning rate limit the amount of activated acid produced as well as its diffusion within the stamp.

**Figure 4:**
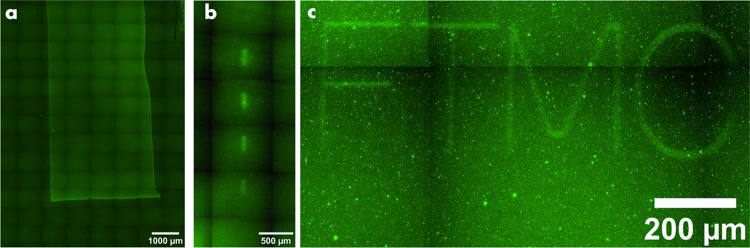
Repeated deprotection using a reusable, regenerated stamp. (a) Whole area deprotection. Rectangular (b) and letter (c) patterns produced using the PDMS stamp from (a).

We also attempted to repeatedly use the PA-enriched stamp (after regeneration by storing in dark conditions) for deprotection relying on the reversible nature of the merocyanine–spiropyran isomerization process. We found that depending on the power used, some degree of irreversible change was induced by laser illumination, leading to the degradation of the PA component in the stamp and therefore diminished deprotection efficiency upon repeated use. Figure 4 shows the fluorescence images of the separate SAM samples, which have been deprotected using the same PA-enriched stamp.

The first sample was deprotected by exposure to the unfocused illumination from the blue LED with a power density of 2.76 mW cm^–2^, which resulted in the predictable deprotection in the area of contact with the stamp (Figure 4a). The second sample was processed by scanning the focused BLD beam in the line fashion using the same stamp after the regeneration in dark conditions (24 h) (Figure 4b). While the fluorescence image reveals that the deprotection of primary amines still took place, the fluorescence intensity is lower than in the comparable experiment with the freshly prepared PA-enriched stamp, indicating a reduced deprotection efficiency. Most likely, chemical degradation of PA takes place at high illumination power densities. On the other hand, under similar conditions in an organic solvent merocyanine photoacids show very high stability and switching robustness as probed by NMR spectroscopy.^58^ The only detectable minor side products after repeated photo cycling are the hydrolysis products, resulting from the reaction with trace water. Such a degradation pathway is less plausible in the PDMS stamp where no or very little water is present. Moreover, Liao and co-workers showed that structurally similar PA possesses high thermal and photochemical stability in poly(hydroxyethyl methacrylate).^59^ Although the exact degradation pathway remains unclear, the photoinduced reaction of PA with residual silanes in the PDMS matrix is another viable possibility for its inactivation (Figure S12). This is strongly supported by the fact that PA covalently fixed within the polyacrylate polymer chain is perfectly stable during long photocycling.^60,61^ Such a covalent fixation of PA in the polymer matrix in the second-generation stamps would also allow mitigation of the diffusion spread and a further increase in patterning resolution.

## Conclusions

We have reported proof-of-principle studies on a new surface patterning technique that utilizes a highly affordable experimental setup and molecular components to achieve chemically reactive features with a resolution of several microns. We have shown that PDMS stamps doped with reversible merocyanine photoacid effectively transfer patterns generated by conventional HD-DVD laser on Boc-functionalized SAMs. In contrast to the majority of photolithographic techniques, the method invented herein requires no mask, allowing direct on-demand writing. Under optimal conditions, a resolution of better than 10 μm is achieved. Preliminary experiments also revealed a high potential of our method for multiple uses of the stamp. Some degradation of the photoacid and broadening of the pattern are observed due to chemical reactivity and diffusion of the photoacid, respectively; however, these obstacles most likely can be eliminated in the second generation of polymeric stamps comprising covalently attached photoacid. The use of a visible-light source, as implemented in the commercial inexpensive HD-DVD/Blu-ray hardware as a writing tool is yet another hallmark of our method, which will aid its wider future applications in surface science.

## Supporting Information Available

Description of HD-DVD-based photochemical patterning setup, characterization of the laser beam, and control experiments (varying irradiation intensity, visualization using avidin, reusable stamp experiment, photoacid reaction with the curing agent) (PDF)
