# Supplementary material for: Maskless, Reusable Visible-Light Direct-Write Stamp for Microscale Surface Patterning
Source: ACS Appl Mater Interfaces. 2023 Feb 17;15(8):11259–67. doi: 10.1021/acsami.2c20568 (PMC11008783; doi:10.1021/acsami.2c20568)
Supplement: Supplementary file 1 — am2c20568_si_001.pdf [file am2c20568_si_001.pdf]

Supporting Information

Maskless, Reusable Visible-Light Direct-Write Stamp for Microscale Surface Patterning

Tomas Javorskis,[a] Tomas Rakickas,[a] Alberta Jankūnaitė,[b] Šarūnas Vaitekonis,[a] Artūras Ulčinas[a] and Edvinas Orentas*[a,b]*

[a] Department of Nanoengineering, Center for Physical Sciences and Technology, Savanorių 231, LT-02300 Vilnius, Lithuania

[b] Department of Organic Chemistry, Vilnius University, Naugarduko 24, LT-03225 Vilnius, Lithuania

Table of Contents

Materials and methods 3

Materials 3

VIS spectra of photoacid-enriched PDMS stamp 3

Experimental details of the patterning experiments 3

Characterization of the blue laser beam 4

Boc deprotection using photoacid-enriched PDMS and focused BLD illumination (varying intensity experiment) 5

Boc deprotection using photoacid-enriched PDMS and focused BLD illumination (dots experiment)............ 6

Boc deprotection using photoacid-enriched PDMS and focused BLD illumination (visualization using avidin)...... 6

Boc deprotection using photoacid-enriched, microstructured PDMS stamp and focused BLD illumination 7

Boc deprotection using photoacid-enriched PDMS and blue light (reusable stamp experiment) 7

Experimental details and results of the control experiments 8

Effect of photoacid-enriched PDMS stamp compared to PDMS-only stamp 8

Effect of photoacid-enriched PDMS stamp without illumination 8

Effect of BLD illuminated photoacid-enriched PDMS stamp compared to BLD illumination-only (no stamp)........ 9

Effect of initial biotin treatment step in Qdot 655 protocol 10

AFM characterization of the microstructured photoacid-enriched PDMS stamp 10

Fluorescence image analysis procedure 10

Photo-induced chemical reaction between the photo-acid and the curing agent............ 11

References 11

Materials and methods

Materials

Compounds used for the preparation of self-assembled monolayer (thiols), photoacid, derivatization reagent (Biotin-PEG4-NHS) and their chemical structures are given in Figure S1.

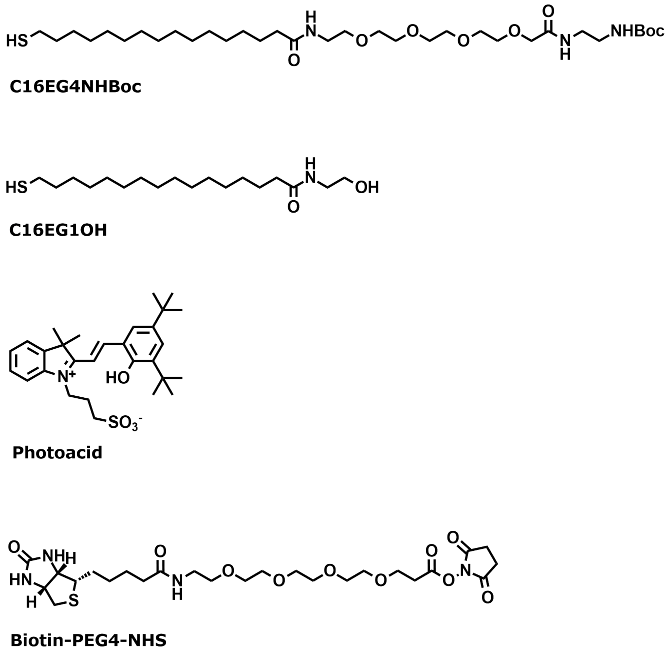

Figure S1. Chemical structures and names of compounds used in the patterning and control experiments.

VIS spectra of photoacid-enriched PDMS stamp

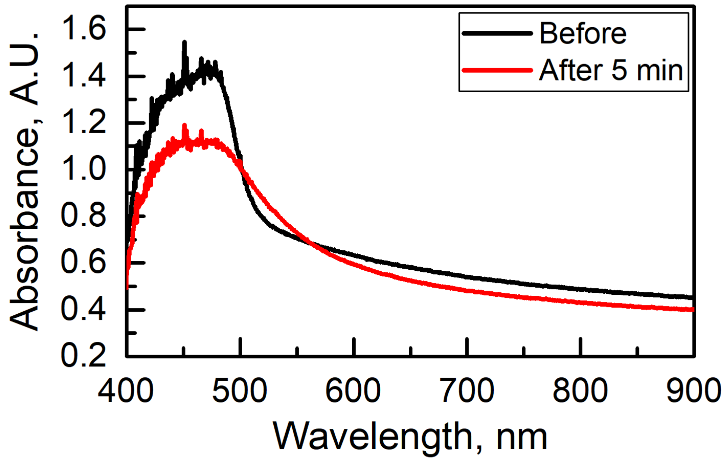

Figure S2. Absorption spectra of the photoacid-enriched stamp before illumination with the blue light (black curve) and after 5 minutes of illumination (red curve).

Experimental details of the patterning experiments

HD-DVD-based photochemical patterning setup

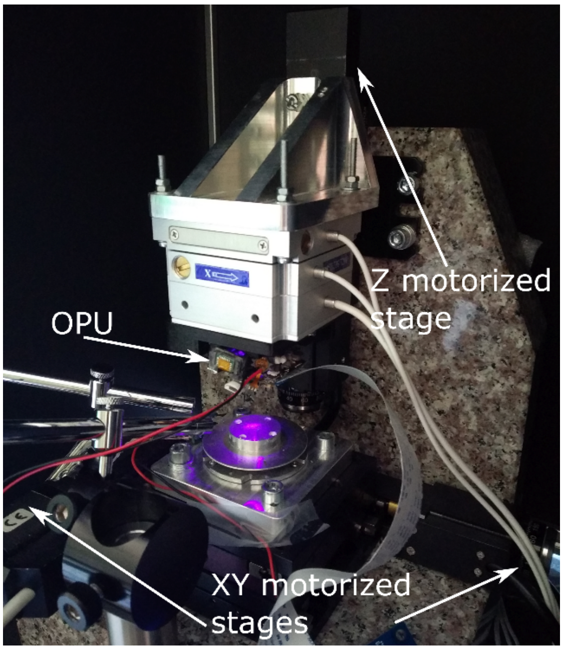

Figure S3. Photograph of the main parts of the experimental setup based on HD-DVD optical pickup (OPU).

Briefly, experimental setup was built around the HD-DVD optical pickup (OPU; PHR-803T, Toshiba, Japan). This OPU contains two laser diodes emitting in three visible wavelength regions: one for infra-red (780 nm) and red (650 nm), and one for blue (405 nm). As the absorbance of the photoacid used in this work is located primarily in the blue region, only the blue laser diode was used. OPU has an integrated lens with numerical aperture of 0.85 which allows to focus the laser beam to nearly diffraction-limited spot size, eliminating the need for any further optics. The lens may be actuated by the voice coils for focusing (vertical direction) and positioning in one planar axis (lateral direction). Astigmatic detection system for tracking of the focal position[1] is also incorporated into the OPU. Excellent review of the characteristics of the various DVD and Blu-ray OPUs and repurposing for research is given by Hwu et al.[2]

In our setup, the OPU is attached to the custom 3D-printed plastic holder which in turn is fixed to the custom aluminum right-angle bracket (the assembly can optionally include a three-dimensional nanopositioning stage) and mounted on the motorized vertical (Z) positioning stage (8MT30-50, Standa, Lithuania). Sample positioning in XY was implemented by two motorized positioning stages (8MT167-25, Standa, Lithuania). All motorized stages were computer-controlled by software developed in-house.

The OPU is controlled by in-house developed and built electronic controller. Controller allows to track the beam focusing on the surface, and to set the intensity of the laser diodes by means of either laser current source integrated in OPU or dedicated constant current supply circuit integrated in the controller board. Detailed description of the experimental workstation will be reported in future publication.

Characterization of the blue laser beam

Optical power of the blue laser diode (BLD) was measured using the power meter (PD300-TP, Ophir Photonics, Israel) and used for calibrating the BLD controller.

To estimate the profile of the BLD beam at the focus, a knife-edge approach was used. BLD beam was focused and scanned on the transparent glass sample with square-shaped Cr microstructures through the overlaid photoacid-enriched PDMS stamp. The Cr square line corrugations were close to or below the diffraction limit thus allowing to use it as a knife-edge. Figure S4 shows the typical profile of the signal produced by the ADS while scanning the square microstructure in X direction. By analyzing profiles in both scanning directions, the dimensions of the beam were estimated to be 0.47±0.2 μm and 0.38±0.2 μm in X and Y directions, respectively.

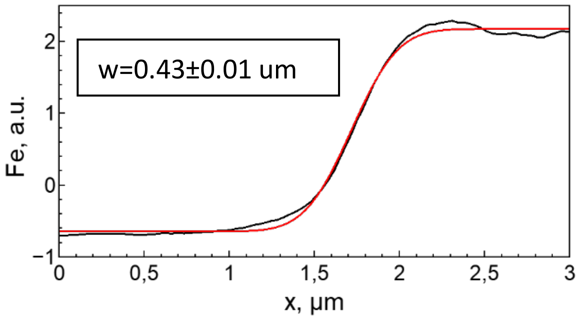

Figure S4. Typical profile of the ADS signal (black line) as the focused beam is scanned on the Cr microstructure serving as knife-edge, and a fit (red line); w – fitted beam width at focus.

Control experiments and photochemical experiments without patterning were conducted by illuminating the sample by blue (440 nm) LED with optical power density of 2.76 mW/cm^2. For direct comparison, the values of optical power density for BLD illumination given below were estimated for the thickness of the photoacid-enriched PDMS layer of 30 μm and assuming negligible absorption and scattering in the supporting PDMS layer.

Boc deprotection using photoacid-enriched PDMS and focused BLD illumination (varying intensity experiment)

Photoacid-enriched PDMS stamp was placed on 5% C16EG4NHBoc/95% C16EG1OH SAM. First, the BLD beam was focused on the gold film surface using OPU ADS. Then, the sample was scanned in line fashion for 200 μm (25 repetitions per line) with varying scan rate (25 – 200 μm/s) while illuminating it with focused BLD beam (optical power 0.08– 0.82 mW; equivalent power density 5.0 – 51.6 W/cm^2). Qdot545 treatment and fluorescence imaging were carried out to visualize the deprotected areas. Figure S5 shows the images obtained in the case of 25 μm/s scan rate with 28.9 W/cm^2 BLD power density (upper part) and 51.6 W/cm^2 (lower part), together with respective fluorescence intensity line profiles.

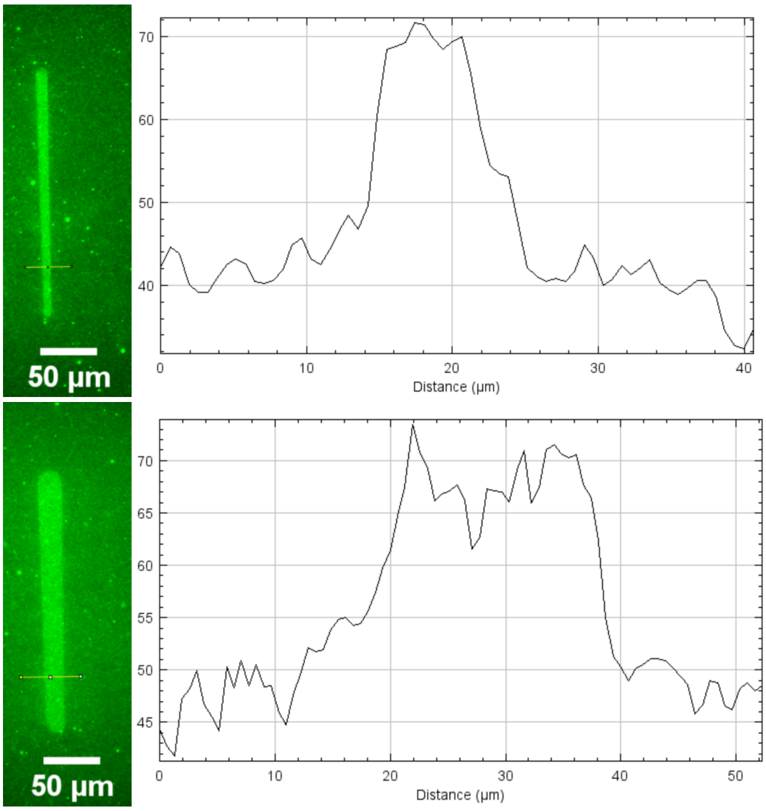

Figure S5. Boc deprotection using photoacid-enriched PDMS stamp and line-scanned focused BLD illumination; top panel- 28.9 W/cm^2 ; bottom panel - 51.6 W/cm^2 optical power density.

Boc deprotection using photoacid-enriched PDMS and focused BLD illumination (dots experiment)

Photoacid-enriched PDMS stamp was placed on 5% C16EG4NHBoc/95% C16EG1OH SAM. Next, illumination with stationary focused BLD beam was performed at varying optical power (0.08, 0.46, 0.81 and 0.82 mW; equivalent power density 5.0, 28.9, 50.9 and 51.6 W/cm^2) and time (2, 4, 10, 20 and 50 s). Qdot545 treatment and fluorescence imaging were carried out to visualize the deprotected areas. Figure 2c in the manuscript shows the obtained fluorescence images.

Boc deprotection using photoacid-enriched PDMS and focused BLD illumination (visualization using avidin)

Photoacid-enriched PDMS stamp was placed on 5% C16EG4NHBoc/95% C16EG1OH SAM. Then, the sample was scanned in line fashion for 200 µm (1 repetition per line) with varying scan rate (1.25 – 5 µm/s) while illuminating it with focused BLD beam (optical power density 28.9 and 51.6 W/cm^2). Qdot655 treatment and fluorescence imaging were carried out to visualize the deprotected areas. Figure S6 shows the fluorescence images for the BLD illumination with 28.9 W/cm^2 (top panel) and 51.6 W/cm^2 (bottom panel) optical power density.

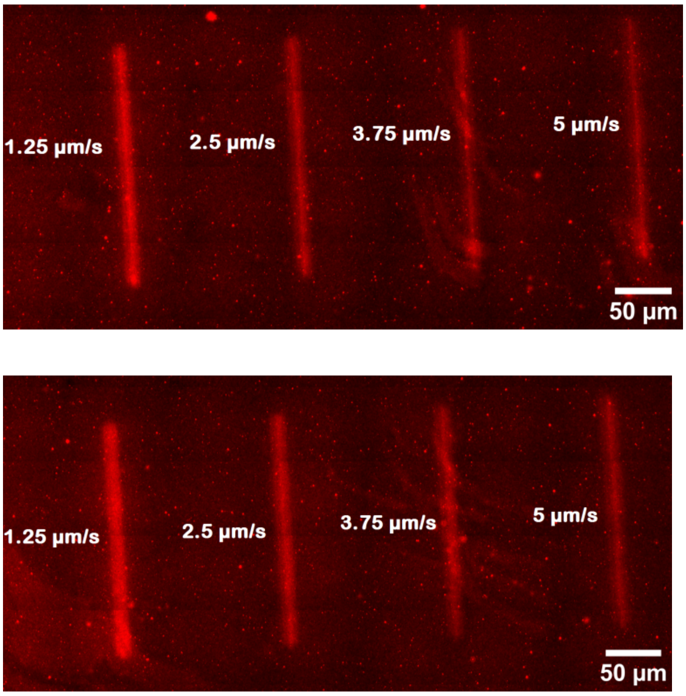

Figure S6. Boc deprotection using photoacid-enriched PDMS stamp and line-scanned focused BLD illumination; avidin/Qdot655 visualization; optical power density - 28.9 W/cm^2 (upper panel); 51.6 W/cm^2 (lower panel).

Boc deprotection using photoacid-enriched, microstructured PDMS stamp and focused BLD illumination

Photoacid-enriched, Klarite-microstructured PDMS stamp was placed on 5% C16EG4NHBoc/95% C16EG1OH SAM. Then, the sample was scanned in line fashion for 200 µm (25 repetitions per line) with varying scan rate (25 – 200 µm/s) while illuminating it with focused BLD beam (optical power density 28.9 W/cm^2). Qdot545 treatment and fluorescence imaging were carried out to visualize the deprotected areas. Figure 3d in the manuscript shows the obtained fluorescence image, revealing the patterns composed of square-shaped segments with dimensions corresponding to the Klarite microstructures.

Boc deprotection using photoacid-enriched PDMS and blue light (reusable stamp experiment)

Photoacid-enriched PDMS stamp was placed on 5% C16EG4NHBoc/95% C16EG1OH SAM. Whole sample was illuminated with blue LED for 0.5 h. Qdot545 treatment and fluorescence imaging were carried out to visualize the deprotected area (Figure 4a in the manuscript). Next, the same used stamp was left in dark conditions overnight and washed with ethanol before next application on freshly prepared SAM sample. For repeated stamp use, several rectangular scans (200x50 µm) of focused BLD beam (51.6 W/cm^2) were carried out. Qdot545 treatment and fluorescence imaging revealed the scanned patterns (Figure 4b in the manuscript). While the efficiency of deprotection as evaluated from the fluorescence intensity is diminished upon repeated use, using the regenerated stamp allows to achieve the degree of deprotection comparable to the activation with blue LED.

Experimental details and results of the control experiments

Effect of photoacid-enriched PDMS stamp compared to PDMS-only stamp

Two different stamps were placed side-by-side on 5% C16EG4NHBoc/95% C16EG1OH SAM and illuminated with blue LED for 0.5 h. Subsequent treatment with Qdot545 and fluorescence imaging revealed that the deprotection has taken place only at the area where photoacid-enriched stamp has been placed, confirming the selectivity due to the action of the activated photoacid (Figure S7).

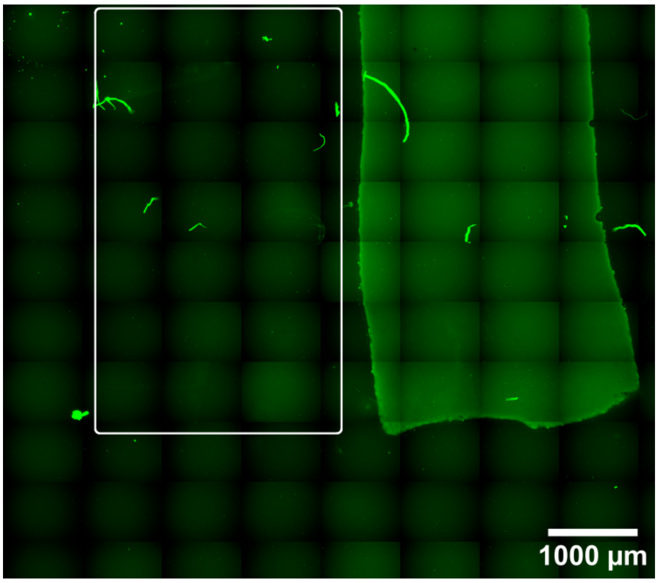

Figure S7. Boc deprotection using photoacid-enriched PDMS stamp (right) vs PDMS stamp (outline represents position of the stamp, left).

Effect of photoacid-enriched PDMS stamp without illumination

Photoacid-enriched PDMS stamp was placed on 5% C16EG4NHBoc/95% C16EG1OH SAM. The sample with the stamp was kept in the dark conditions and heated to ~40 ^{o}C for 0.5 h. Heating was intended to simulate any possible increase in the temperature of the stamp when performing experiment with blue LED illumination. After treatment with Qdot545 and fluorescence imaging no Boc deprotection was observed (Figure S8).

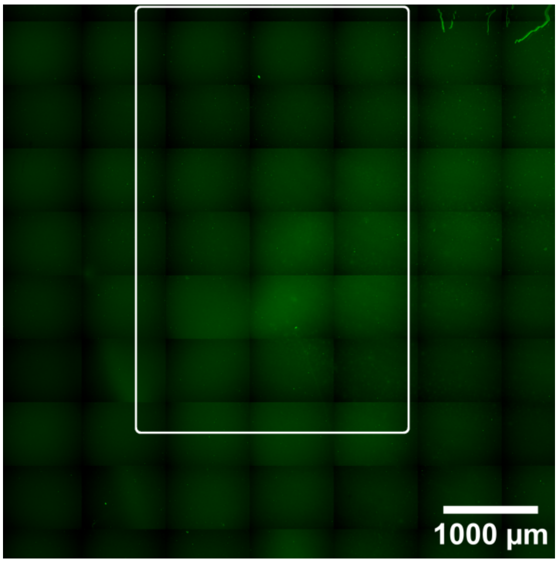

Figure S8. Effect of photoacid-enriched PDMS stamp in the dark conditions with moderate heat (outline represents position of the sample placement). No deprotection is observed.

Effect of BLD illuminated photoacid-enriched PDMS stamp compared to BLD illumination-only (no stamp)

Two separate experiments were performed:

1) Photoacid-enriched PDMS stamp was placed on 5% C16EG4NHBoc/95% C16EG1OH SAM. Area of 200x50 µm was scanned using focused BLD illumination with scan rate of 105 µm/s and optical power density of 51.6 W/cm^2. After treatment with Qdot655 and fluorescence imaging Boc deprotection was observed in the scanned area (Figure S9, left panel).

2) The same scanning and illumination conditions were used with the substrate having 5% C16EG4NHBoc/95% C16EG1OH SAM; no stamp has been applied. After treatment with Qdot655 and fluorescence imaging no Boc deprotection was observed in the scanned area (Figure S9, right panel), confirming that the BLD illumination alone does has no significant effect on the SAM or protective group.

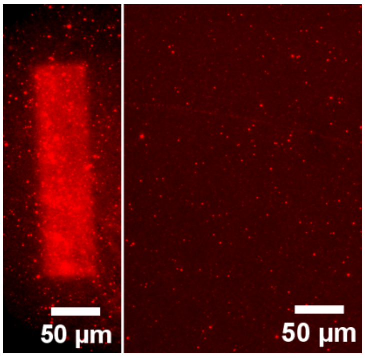

Figure S9. Boc deprotection using photoacid-enriched PDMS stamp (left) vs no stamp (right).

Effect of initial biotin treatment step in Qdot 655 protocol

The goal of the following experiment was to check if omitting the initial N-hydroxysuccinimide-activated biotin linker treatment step in Qdot655 protocol will lead to the visualization of the illuminated area, which would signify the false positive, as avidin should not specifically bind to free amines. Photoacid-enriched PDMS stamp was placed on 5% C16EG4NHBoc/95% C16EG1OH SAM and focused BLD beam was scanned with varying scan rate (1.25–105 μm/s at 51.6 W/cm^2) for 200 μm. Treatment with Qdot655 was performed without initial biotin step. No fluorescece was observed in the scanned area, confirming the specificity of Boc deprotection and the absence of significant damage to SAM, which would lead to the non-specific attachment of avidin.

AFM characterization of the microstructured photoacid-enriched PDMS stamp

AFM was used to confirm that the Klarite mold was adequately reproduced in the photoacid-enriched PDMS replica. AFM scans were done on NanoWizard 3 AFM (Bruker, USA) in intermittent contact mode in air using Scout 350 probes (Nunano, UK). Figure S10 shows stamp topography representations, confirming that the photoacid-enriched stamp surface contains the array of the pyramidal microstructures with pitch of around 2 μm and height of 800 nm.

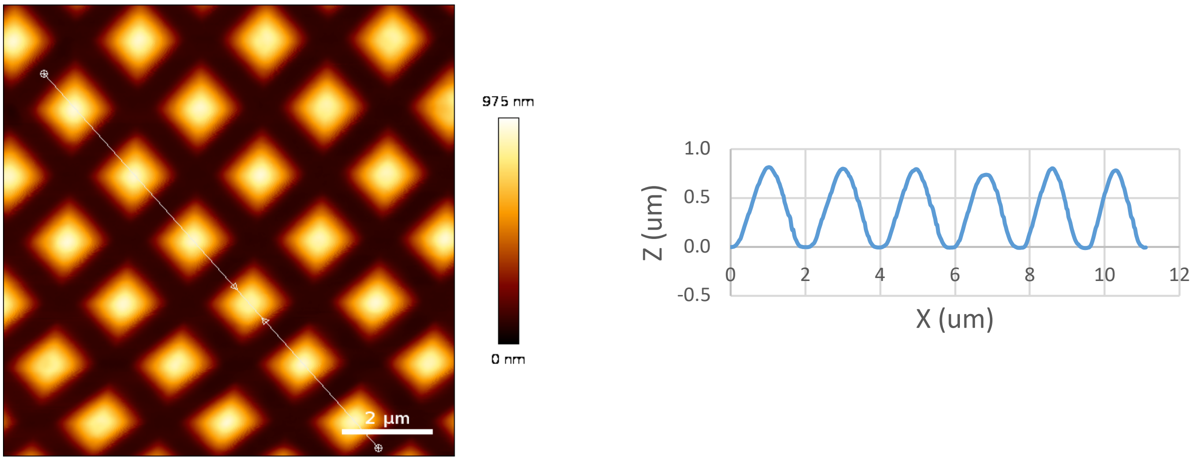

Figure S10. Atomic force microscopy of the microstructured photoacid-enriched PDMS stamp. (left) height image; (right) height cross-section along the line shown in topography image.

Fluorescence image analysis procedure

ImageJ (software version 1.53t)[3] was used to extract dot area, mean fluorescence and fluorescence background values shown in Figure 2d,e. Threshold function (default method) was applied to the corresponding fluorescence image and particle analysis routine was run while restricting minimum detectable grain area to 50 μm^2. Figure S11 shows the contours of the detected grains (in cyan) and detected grain area (in red) overlaid on the original fluorescence image for I=28.9 W/cm^2.

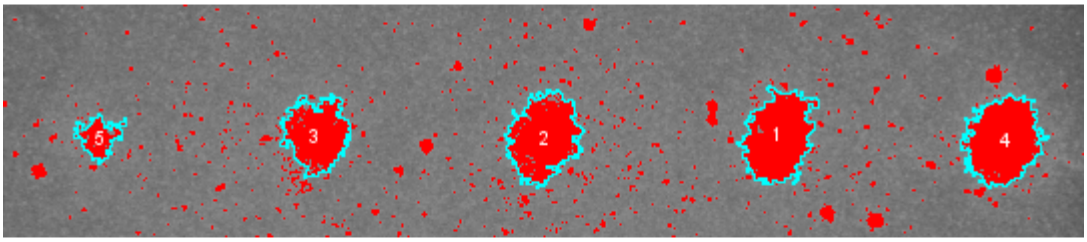

Figure S11. Image representing dot outline detection for area and fluorescence intensity calculations for values presented in Figure 2d,e.

Photo-induced chemical reaction between the photo-acid and the curing agent

To demonstrate the mechanism of reduced regeneration efficiency of the PA in the presence of PDMS curing agent and under blue light irradiation, the control experiment was performed by mixing PA (5.0 mg) and curing agent (50 µL) in dry 1,2-dichloroethane. The so-obtained solution was irradiated with blue light (LED, 20 W, λ = 450 nm) for 30 min. The color of the solution changes irreversibly from orange to yellow indicating the chemical reaction (Figure S12).

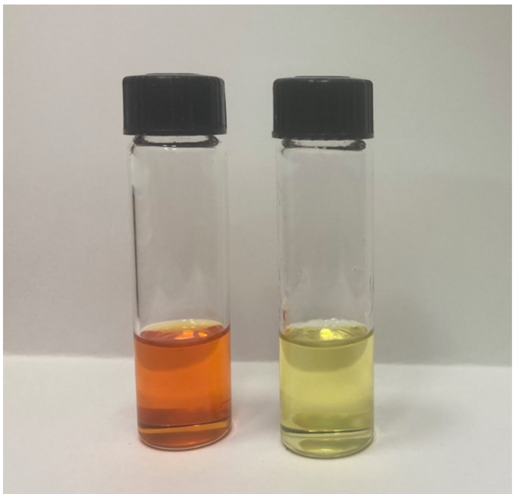

Figure S12. A solution of PA (left) and PA+ curing agent (right) after irradiation with blue LED.

References

(1) Shimomura, T.; Izawa, C.; Matsui, T. Development of a Compact Optical System for Microarray Scanning Using a DVD Pickup Head. *Review of Scientific Instruments* **2008**, *79* (3), 035101-035101–035106. https://doi.org/doi:10.1063/1.2885609.

(2) Hwu, E. E.-T.; Boisen, A. Hacking CD/DVD/Blu-Ray for Biosensing. *ACS Sens.* **2018**. https://doi.org/10.1021/acssensors.8b00340.

(3) Schindelin, J., Arganda-Carreras, I., Frise, E., Kaynig, V., Longair, M., Pietzsch, T., … Cardona, A.. Fiji: an open-source platform for biological-image analysis. *Nature Methods*, *9*(7) (2012) 676–682. https://doi.org/10.1038/nmeth.2019
